# Optimised Deep Learning for Gastrointestinal Polyp Classification: A Controlled Benchmark of Five CNN and Transformer Architectures with Grad-CAM Interpretability

**DOI:** 10.3390/diagnostics16142182

**Published:** 2026-07-13

**Authors:** Zhengsui Gu, Hoda Anwar Ibrahim, Wamadeva Balachandran, Md Nazmul Huda

**Affiliations:** 1 School of Communication and Information Engineering, Chongqing University of Posts and Telecommunications, Chongqing 400065, China; zhengsui.gu@cqupt.edu.cn; 2 Department of Engineering, College of Engineering, Brunel University of London, Uxbridge UB8 3PH, UK; hoda.ibrahim@brunel.ac.uk (H.A.I.); wamadeva.balachandran@brunel.ac.uk (W.B.)

**Keywords:** colorectal cancer, polyp classification, deep learning, convolutional neural networks, Vision Transformer, Grad-CAM, transfer learning, Kvasir dataset, gastrointestinal endoscopy

## Abstract

**Background/Objectives:** Colorectal cancer (CRC) is the second leading cause of cancer-related mortality worldwide, with polyp miss rates of up to 26% reported during colonoscopy and classification accuracy remaining highly operator-dependent. Accurate multi-class polyp subtype classification is clinically critical, as it directly determines treatment decisions: adenomatous polyps require resection, whereas hyperplastic lesions may warrant only surveillance. This study aims to systematically compare five deep learning architectures for five-class gastrointestinal polyp classification and to provide clinically interpretable diagnostic insights through Grad-CAM visualisation. **Methods:** ResNet50, VGG16, EfficientNet-B3, DenseNet121, and Vision Transformer (ViT-B/16) were evaluated on the Kvasir Dataset V2 (5000 images, five classes) under a unified training and evaluation protocol on common GPU hardware. All models employed ImageNet transfer learning with a redesigned multi-layer classification head. Two optimisation strategies were applied: SGD with cosine annealing for CNN architectures, and AdamW with linear warmup for ViT-B/16. Gradient-weighted Class Activation Mapping (Grad-CAM) was applied to generate spatial attention heatmaps for qualitative clinical interpretation. **Results:** Under a single 80/10/10 split, ViT-B/16 attained the highest accuracy (97.2%); however, because a single split is sensitive to sampling, the evaluation was strengthened with stratified five-fold cross-validation (mean ± SD). Under cross-validation, EfficientNet-B3 achieved the highest accuracy at 95.90 ± 0.35%, followed closely by ViT-B/16 (95.12 ± 0.72%), then ResNet50 (91.74 ± 0.74%), DenseNet121 (90.32 ± 0.70%), and VGG16 (88.50 ± 1.72%); the small standard deviations indicate that all models, including ViT-B/16, were stable across folds. Pairwise McNemar tests with Holm correction found that every difference was statistically significant (*p* < 0.05), including the EfficientNet-B3 advantage over ViT-B/16 (*p* = 0.010). ViT-B/16 thus remained a strong, stable performer that significantly outperformed the three remaining CNNs, while the cross-validated ranking placed the most compact model, EfficientNet-B3, first: a Vision Transformer was highly competitive with, but not superior to, the strongest CNN. A consistent, architecture-agnostic misclassification pattern was identified between dyed-lifted polyps and dyed-resection margins across all five models, consistent with a task-level visual ambiguity that may also reflect overlapping class definitions and annotation factors, with direct clinical implications. Grad-CAM analysis, quantified by attention-entropy and concentration metrics, showed that model attention remained focused on relevant stained tissue regardless of whether predictions were correct, indicating that the dyed-class confusions reflect genuine visual ambiguity rather than a localisation failure. **Conclusions:** Under cross-validation, EfficientNet-B3 achieved the highest accuracy on the Kvasir V2 five-class task, significantly outperforming all other architectures, with ViT-B/16 being a close and competitive second. The identified confusion between post-procedural chromoendoscopic classes is unlikely to be fully resolved by architectural changes alone and may require higher-resolution imaging or domain expert re-annotation. These findings contribute to the evidence base for explainable deep learning in gastrointestinal endoscopy; external, multi-centre validation remains necessary before clinical adoption.

## 1. Introduction

Colorectal cancer (CRC) ranks as the second leading cause of cancer-related mortality globally, accounting for over 900,000 deaths annually as of 2023 statistics [1]. A well-established pathological sequence, the adenoma–carcinoma pathway, accounts for 70–80% of CRC cases, in which adenomatous polyps serve as the principal precursor lesion [2]. Critically, timely endoscopic identification and resection of polyps before malignant transformation can dramatically reduce CRC incidence and mortality; it is estimated that 75% of CRCs originate from adenomatous polyps, making accurate polyp characterisation central to clinical preventive strategies.

Colonoscopy remains the gold standard for colorectal polyp screening; however, its diagnostic quality is highly operator-dependent. Miss rates during colonoscopy range from 2% to 26%, and inter-observer agreement among international expert endoscopists in morphological classification reaches only approximately 85% [2]. These limitations are particularly pronounced for small (≤5 mm) and flat polyps, which are challenging to identify under standard white-light or narrow-band imaging without extensive experience. Furthermore, the pathological nature of polyps directly determines clinical management: adenomatous polyps require resection, whereas hyperplastic polyps may only warrant surveillance, making accurate subtype classification a direct driver of treatment decisions.

Deep learning (DL) has demonstrated substantial promise in medical image analysis, including polyp detection, segmentation, and classification. The Kvasir Dataset V2 [3], curated by the Simula Research Laboratory, has established itself as a key benchmark for reproducible endoscopy AI research, supporting a growing body of work on polyp detection, segmentation, and classification [4,5,6,7]. However, multi-class polyp classification that discriminates between pathological subtypes remains less studied than binary polyp detection or pixel-level segmentation. Recent benchmarking by Khalafi et al. shows that even advanced vision-language models (VLMs) such as GPT-4.1 achieve a weighted F1-score of only 55.07% on polyp classification, compared to 74.94% for ResNet50 under equivalent conditions [8], underscoring the persistent difficulty of fine-grained endoscopic categorisation.

### 1.1. Research Gap, Aim, and Objectives

Despite extensive work on polyp detection and segmentation, controlled multi-class classification studies that compare several architectures under identical conditions, quantify the statistical significance of their differences, and pair the results with interpretability analysis remain scarce. Reported comparisons are frequently confounded by differing training budgets, optimisers, input resolutions, or hardware, which makes architecture-level conclusions difficult to substantiate.

The aim of this study is therefore to provide a rigorous, reproducible, and clinically interpretable benchmark of representative convolutional and Transformer architectures for five-class gastrointestinal image classification. The specific objectives are:

- To evaluate five widely used architectures (ResNet50, VGG16, EfficientNet-B3, DenseNet121, and ViT-B/16) on the Kvasir V2 five-class task under a unified training and evaluation protocol on common hardware;
- To quantify the robustness of the comparison using cross-validation and to test whether inter-model differences are statistically significant;
- To characterise the accuracy–efficiency trade-off of each architecture using comparable complexity and runtime measures;
- To apply Grad-CAM-based interpretability across all architectures to identify and explain clinically relevant misclassification patterns.

### 1.2. Contributions

This paper makes the following contributions:

1. **A controlled five-architecture benchmark on five-class Kvasir V2:** To our knowledge, these five architectures have not previously been compared under a single, identical protocol on the Kvasir V2 five-class task [9,10]. Existing comparisons either restrict evaluation to binary classification, use fewer than five architectures, or employ different datasets.
2. **Statistically validated evidence that a Vision Transformer is competitive with, but not superior to, the strongest CNN:** Under cross-validation with McNemar significance testing, EfficientNet-B3 attains the highest accuracy and significantly outperforms all other models (p<0.05), while ViT-B/16 ranks a close second and significantly outperforms the remaining CNNs. This shows that a Vision Transformer is highly competitive on this task, tempering the assumption that Transformers necessarily underperform CNNs on small medical datasets [11], without overstating Transformer superiority.
3. **Unified multi-layer classification head for fair cross-architecture comparison:** A redesigned head incorporating dropout regularisation and batch normalisation is applied identically to all five backbones, reducing head-design confounds in the comparison.
4. **Cross-architecture Grad-CAM interpretability analysis:** Grad-CAM is applied systematically across all five architectures, identifying a clinically relevant, consistent misclassification pattern between dyed-lifted polyps and dyed-resection margins, which we examine in relation to both model behaviour and dataset characteristics.
5. **Comprehensive literature benchmarking:** The results are contextualised against published work on Kvasir V2 and related gastrointestinal datasets, noting that all five evaluated models exceed the published DFE-IANet baseline (93.94%) [12], while flagging differences in class count and dataset composition that qualify such comparisons.

**Figure 1:**
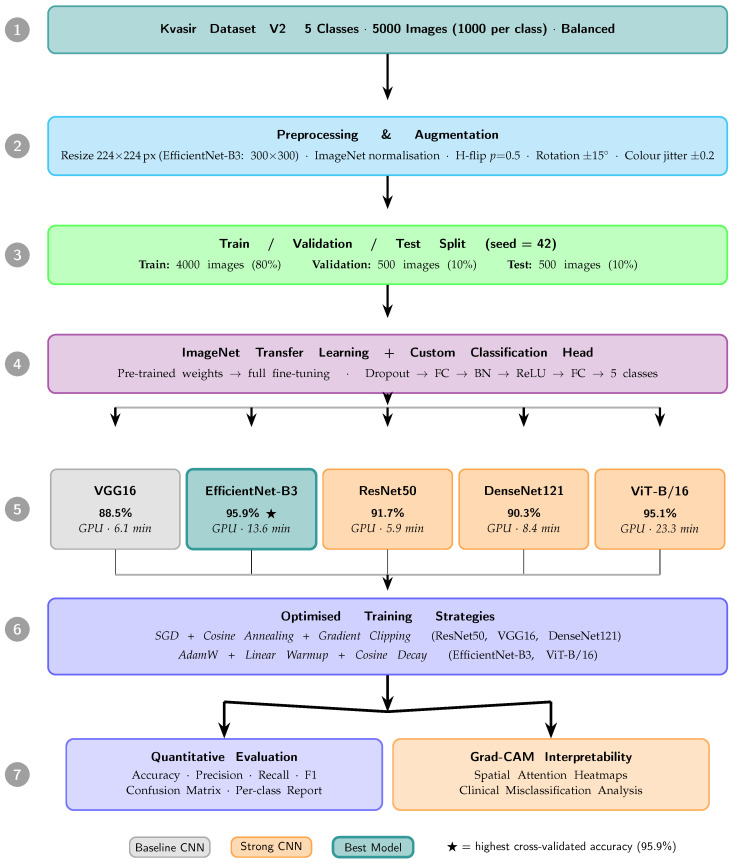
End-to-end pipeline for five-class gastrointestinal polyp classification on the Kvasir Dataset V2. Numbered stages 1–7 describe the complete workflow. Model boxes report test accuracy and training time; colour encodes architecture category (grey: baseline CNN, orange: strong CNN, teal: best model).

The complete experimental pipeline is illustrated in Figure 1. The remainder of this paper is organised as follows: Section 2 reviews the related work. Section 3 describes the dataset, preprocessing, architectures, and training configuration. Section 4 presents the experimental results. Section 5 discusses the findings in context, and Section 6 concludes the paper.

## 2. Related Work

### 2.1. Clinical Evidence for AI-Assisted Colonoscopy

Beyond focused technical benchmarks, a substantial clinical literature has evaluated AI-assisted colonoscopy in real-world and randomised settings. Early real-time CNN systems demonstrated polyp localisation and identification accuracy of 96% during live screening colonoscopy [13], and computer-aided classification of diminutive polyps has been shown to support optical biopsy decisions with high diagnostic accuracy [14]. These detection-stage results have since been corroborated in prospective and randomised trials: a multicentre randomised trial reported that real-time computer-aided detection significantly increased the detection of colorectal neoplasia compared with standard colonoscopy [15]. Ahmad et al. reviewed the current evidence base and future directions for artificial intelligence and computer-aided diagnosis in colonoscopy more broadly [16], while an initial clinical deployment study reported strong early performance for AI-assisted polyp detection under routine conditions [17]. Beyond detection accuracy, an add-on economic analysis of a randomised trial estimated meaningful cost savings from AI-aided polyp diagnosis through reduced unnecessary pathological review [18]. At a broader level, Srivastava et al. surveyed deep learning approaches across the gastrointestinal cancer diagnostic pipeline [19], and Pacal et al. reviewed deep learning applications specifically in colon cancer [20]. Collectively, this body of work establishes strong clinical motivation for AI-assisted colonoscopy but concentrates on lesion detection rather than fine-grained pathological subtype classification, the focus of the present study.

### 2.2. Polyp Detection and Segmentation

The dominant body of DL research in gastrointestinal endoscopy has targeted binary polyp detection and pixel-level segmentation. U-Net variants have been widely adopted for polyp segmentation, benefiting from skip connections that preserve spatial resolution [4,6]. More recently, Transformer-based architectures have been incorporated into segmentation pipelines, with ConvNeXt backbones demonstrating consistent improvements over ResNet50, VGG16, and standard ViT on multiple benchmark datasets [6,7]. Despite strong segmentation performance, these models are not directly applicable to multi-class pathological classification, which requires discriminating between polyp subtypes rather than delineating polyp boundaries.

### 2.3. Polyp Classification with CNNs

Multi-class polyp classification has received comparatively limited attention. Wang et al. introduced DFE-IANet, a dual-domain feature extraction network with interaction attention, achieving a Top-1 accuracy of 93.94% on the Kvasir dataset [12]. Houmaidi et al. evaluated VGG16 and complementary architectures on a four-class Kvasir V2 subset of 4000 images, reporting a best accuracy of 96.5% [21]. Wahid et al. assessed interpretable CNN-based approaches on the eleven-class HyperKvasir dataset, obtaining accuracy in the range 80–83% [22]. Krenzer et al. explored few-shot learning for polyp classification under limited-data conditions, demonstrating that standard architectures can be effectively adapted to small clinical datasets [5]. Neamah et al. [9] compared nine CNN models, including ResNet50, DenseNet201, and EfficientNetV2L, on Kvasir V2, but restricted evaluation to binary classification only. Mazhar et al. [10] compared ResNet50, EfficientNetB2, and ViT-b_32 for polyp classification using only three architectures and a different dataset, without the five-class Kvasir V2 setting.

### 2.4. Vision Transformers and Vision-Language Models

The application of Vision Transformers (ViT) [23] to endoscopic classification remains nascent. Khalafi et al. conducted a head-to-head evaluation of CNN classifiers against VLMs including GPT-4.1, finding that ResNet50 (F1 = 74.94%) substantially outperformed GPT-4.1 (F1 = 55.07%) on polyp classification [8]. This finding motivates the inclusion of ViT-B/16 in the present work as a Transformer-based baseline within the standard supervised fine-tuning paradigm, disentangling the effect of architecture from the large-scale pre-training advantages of VLMs.

### 2.5. Synthesis of Research Gaps

Three gaps emerge from the preceding survey: First, the dominant body of work targets binary detection or pixel-level segmentation rather than fine-grained multi-class subtype classification. Second, existing multi-class comparisons typically evaluate few architectures, or do so under non-identical training budgets, optimisers, resolutions, or hardware, which confounds architecture-level conclusions. Third, interpretability is rarely integrated into such comparisons in a systematic, cross-architecture manner. The present study addresses these gaps by benchmarking five representative architectures under a unified protocol on common hardware, quantifying robustness through cross-validation and statistical testing, and applying Grad-CAM consistently across all models to surface clinically meaningful error patterns. Recent advances in sophisticated medical-image understanding and fine-grained disease classification provide further methodological context for this work. For example, multi-task frameworks for surgical instrument segmentation and depth estimation in minimally invasive surgery [24] and deep learning subtype classification for precision therapeutics [25] illustrate the breadth of sophisticated medical image classification strategies, albeit in different clinical contexts. Within gastrointestinal endoscopy specifically, Shen et al., in *Diagnostics*, combined CNN-based detection with an EfficientNet classifier for multi-hospital polyp detection and classification [26], underscoring both the clinical relevance of this task and the value of rigorous, multi-site evaluation.

## 3. Methodology

### 3.1. Dataset Description

This study employs the Kvasir Dataset Version 2 (Kvasir V2), a publicly available gastrointestinal endoscopy image collection compiled by the Simula Research Laboratory, Norway, and validated by experienced endoscopy hospitals [3]. Five categories were selected: dyed-lifted polyps, dyed-resection margins, normal cecum, polyps, and ulcerative colitis. Although Kvasir V2 contains eight categories, the five selected here are those directly relevant to colorectal polyp and lesion characterisation, which is the clinical focus of this study: they comprise the polyp class, the two post-procedural chromoendoscopic classes that arise during polyp resection (dyed-lifted polyps and dyed-resection margins), a representative pathological mucosal class (ulcerative colitis), and a non-pathological reference class (normal cecum). The remaining Kvasir V2 categories correspond to anatomical landmarks and endoscopic procedure views that fall outside the polyp classification task, and were therefore not included; restricting to these five also yields a balanced 1000-image-per-class problem. A total of 5000 images (1000 per class) were utilised, constituting a balanced five-class dataset. Images were partitioned into training (4000; 80%), validation (500; 10%), and test (500; 10%) sets using stratified random splitting, with a fixed seed of 42 to ensure reproducibility. The selected classes span both pathological (polyps, dyed-lifted polyps, dyed-resection margins, ulcerative colitis) and non-pathological (normal cecum) categories, presenting a clinically realistic multi-class discrimination task.

The class-wise partition is summarised in Table 1. The same per-class balance is preserved within each cross-validation fold (see Section 4).

### 3.2. Data Preprocessing and Augmentation

The preprocessing pipeline comprises the following ordered stages: (1) image decoding; (2) resizing to the architecture-specific input size; (3) conversion to channel-first float tensors; (4) ImageNet mean/standard deviation normalisation; and, for training only, (5) online stochastic augmentation. No additional contrast enhancement or denoising was applied, so the comparison reflects architecture behaviour rather than preprocessing differences.

All images were resized to 224×224 pixels (with the exception of EfficientNet-B3, which was run at its native 300×300 input, as specified by its compound-scaling design [27]) and converted to PyTorch (version 2.8.0, CUDA 12.8) tensors in channel-first format (C×H×W). The 224×224 resolution matches the ImageNet pre-training input of the ResNet, VGG, DenseNet, and ViT-B/16 backbones, which is necessary for effective transfer learning and keeps memory and compute tractable across the five-architecture protocol. The high classification accuracy obtained at this resolution indicates that sufficient discriminative information is retained; nonetheless, the possible loss of fine sub-resolution cues is acknowledged in the Limitations (Section 5) as a factor that may contribute to the residual confusion among post-procedural chromoendoscopic classes, and higher-resolution inputs are identified as future work. Pixel-wise normalisation (Equation (1)) was applied using ImageNet channel statistics:

(1) $$\hat{x}=\frac{x-\mu }{\sigma },\;\mu =\left[0.485,\;0.456,\;0.406\right],\;\sigma =\left[0.229,\;0.224,\;0.225\right],$$

where *x* denotes the per-channel pixel value in [0,1]. Training-time augmentation was applied online without pre-storage, comprising random horizontal flipping (p=0.5), random rotation ($\pm 15^{\circ }$), and colour jitter (brightness, contrast, and saturation perturbations of ±0.2). Validation and test sets received only resizing and normalisation. Because augmentation is applied on-the-fly per epoch rather than by generating and storing additional files, the number of images per epoch remains 4000 (800 per class), as in Table 1; augmentation increases the effective diversity of samples seen during training without altering the class-wise counts or introducing offline dataset expansion.

### 3.3. Network Architectures

Five backbone architectures were evaluated. All were initialised with ImageNet pre-trained weights; the original classifier was replaced with a custom multi-layer head. All backbone parameters were updated jointly with the head during fine-tuning.

#### 3.3.1. ResNet50

ResNet50 [28] introduces identity shortcut connections that enable gradient flow across layers, mitigating vanishing gradients. Its residual mapping is

(2) $$y=F(x,\left\{W_{i}\right\})+x,$$

where F is the learned residual function. The backbone outputs a 2048-dimensional feature vector after global average pooling. The custom head is

$$\begin{array}{cc} \; & \mathrm{Drop}(0.5)\;\to \;\mathrm{FC}(2048\;\to \;1024)\;\to \;\mathrm{BN}\;\to \;\mathrm{ReLU}\\ \; & \;\to \mathrm{Drop}(0.3)\;\to \;\mathrm{FC}(1024\;\to \;512)\;\to \;\mathrm{BN}\;\to \;\mathrm{ReLU}\\ \; & \;\to \mathrm{FC}(512\;\to \;5). \end{array}$$

Grad-CAM targets layer4[-1].conv3 (final residual block, third convolution).

#### 3.3.2. VGG16

VGG16 [29] employs a homogeneous stack of 3×3 convolutions, yielding a 25,088-dimensional flattened feature representation. The custom head maps 25,088→1024, then follows the same structure as ResNet50. Grad-CAM targets features[30] (conv5_3).

#### 3.3.3. EfficientNet-B3

EfficientNet-B3 [27] applies compound scaling across depth, width, and input resolution:

(3) $$d=\alpha^{\phi },\;w=\beta^{\phi },\;r=\gamma^{\phi },\;\alpha \cdot \beta^{2}\cdot \gamma^{2}\approx 2,$$

yielding a 1536-dimensional pooled feature vector. A simplified head (Drop(0.3)→FC(1536→5)) was used, consistent with the EfficientNet design convention. Grad-CAM targets features[-1].

#### 3.3.4. DenseNet121

DenseNet121 [30] connects each layer to all subsequent layers within a dense block, enabling feature reuse and reducing the number of parameters relative to ResNet50. The layer connectivity is

(4) $$x_{\ell }=H_{\ell }\;\left([x_{0},x_{1},\ldots ,x_{\ell -1}]\right),$$

where [·] denotes channel-wise concatenation. The backbone outputs a 1024-dimensional feature vector. The custom head follows the same architecture as ResNet50 (with FC first layer adjusted to 1024→512).

#### 3.3.5. Vision Transformer (ViT-B/16)

ViT-B/16 [23] partitions the 224×224 input into 196 non-overlapping 16×16 patches, projects them into a 768-dimensional embedding space, and processes them through 12 multi-head self-attention layers. The multi-head attention mechanism is

(5) $$\mathrm{Attention}\left(Q,K,V\right)=\mathrm{softmax}\;\left(\frac{QK^{⊤}}{\sqrt{d_{k}}}\right)V,$$

where $d_{k}=64$. The [CLS] token output is used for classification. The head is adapted for Transformer conventions:

$$\begin{array}{cc} \; & \mathrm{LayerNorm}(768)\;\to \;\mathrm{FC}(768\;\to \;512)\;\to \;\mathrm{GELU}\\ \; & \;\to \mathrm{Drop}(0.3)\;\to \;\mathrm{FC}(512\;\to \;5). \end{array}$$

ViT is trained with AdamW and a linear warmup (3 epochs) followed by cosine decay, as Stochastic Gradient Descent (SGD) converges poorly for Transformer fine-tuning [11]. ViT-B/16 is selected as the canonical, widely benchmarked Vision Transformer whose ImageNet pre-training is directly comparable to that of the CNN backbones, enabling a controlled, like-for-like transfer-learning comparison without confounding from architecture-specific pre-training regimes. A broader sweep of Transformer variants (e.g., Swin and DeiT) would further enrich the paradigm-level comparison, and is identified as a topic for future work (Section 5); accordingly, the present conclusions are framed at the level of the specific evaluated models rather than as a general CNN-versus-Transformer claim.

### 3.4. Training Configuration

Two distinct optimisation strategies were employed, with each matched to the convergence characteristics of its architecture family. The assignment was determined by optimiser-family behaviour rather than being chosen arbitrarily: the convolutional backbones used Strategy 1, for which SGD is well established, whereas the attention-based model used Strategy 2, since Transformers are known to converge unstably under plain SGD. EfficientNet-B3, although convolutional, was assigned to Strategy 2 because its compound-scaled mobile-style design benefits from adaptive moment estimation; this assignment is stated explicitly here for transparency.

**Figure 2:**
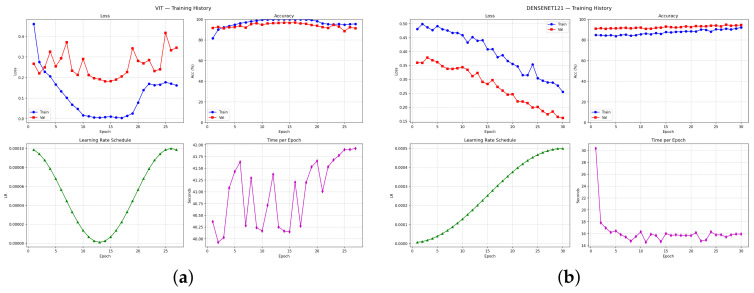
Training and validation loss/accuracy curves for the two GPU-trained architectures. ViT-B/16 converged at epoch 27; DenseNet121 continued to improve steadily throughout 60 epochs. The cosine annealing restart at $T_{\max}=10$ produces a visible improvement step in both curves. (**a**) ViT-B/16 (97.2% single-split test accuracy, 18.4 min on GPU). (**b**) DenseNet121 (95.0% test accuracy, 8.1 min on GPU).

*Strategy 1: SGD with cosine annealing (ResNet50, VGG16, DenseNet121):* ResNet50, VGG16, and DenseNet121 are trained using Stochastic Gradient Descent (SGD) with Nesterov momentum (μ=0.9), weight decay ($\lambda =10^{-4}$), and cosine annealing learning rate scheduling [31]:

(6) $$\eta_{t}=\eta_{\min}+\frac{1}{2}\left(\eta_{0}-\eta_{\min}\right)\;\left(1+\cos\;\frac{\pi t}{T_{\max}}\right),$$

where $\eta_{0}=5\times 10^{-4}$, $\eta_{\min}=5\times 10^{-6}$, and $T_{\max}=10$. The periodic restart at epoch 10 allows the model to escape local minima, and is visible as a performance improvement in the training curves (Figure 2). Gradient clipping (max_norm=1.0) prevents gradient explosion on outlier batches.

*Strategy 2: AdamW with linear warmup and cosine decay (EfficientNet-B3 and ViT-B/16):* Transformer architectures are known to diverge under SGD due to their sensitivity to large early gradient updates [11]. EfficientNet-B3 and ViT-B/16 therefore use AdamW; for ViT-B/16 ($\eta_{0}=10^{-4}$, $\lambda =10^{-2}$) with a 3-epoch linear warmup phase:

(7) $$\eta_{t}=\eta_{0}\cdot \frac{t}{T_{\mathrm{warm}}},\;t\leq T_{\mathrm{warm}},$$

followed by cosine decay to zero. This two-phase schedule stabilises early attention weight formation before gradually reducing the learning rate for fine-grained convergence.

In the original single-split experiment, ResNet50 and VGG16 were trained on an Intel Core CPU platform (16 GB RAM) while EfficientNet-B3, DenseNet121,and ViT-B/16 used a Quadro RTX 6000 GPU (25.2 GB VRAM). In the revised study, all five models were re-trained on the same Quadro RTX 6000 GPU under the cross-validation protocol, so the primary results are free of this hardware disparity.

The base learning rates and schedules above follow the established, literature-recommended settings for each optimiser/architecture family; the remaining hyperparameters (learning rate, weight decay, batch size, and warmup length) were selected by validation-based tuning, choosing the configuration with the highest validation macro-F1. No information from the test set was used for tuning.

To ensure a fair comparison, in the revised study, all five architectures were re-trained and evaluated on the same GPU under a common cross-validation protocol, removing the earlier CPU/GPU disparity. The architecture-appropriate settings that remained (the AdamW schedule for the attention-based model and EfficientNet-B3’s native input resolution) were retained as deliberate, documented choices, and their potential effect on the comparison is discussed in Section 5. Reported training times therefore correspond to identical hardware. On this matched GPU, the mean per-fold training time was 5.9 min for ResNet50, 6.1 min for VGG16, 8.4 min for DenseNet121, 13.6 min for EfficientNet-B3, and 23.3 min for ViT-B/16 (Table 2). EfficientNet-B3 thus reaches the highest accuracy at roughly 1.7× less training time than ViT-B/16, reinforcing its favourable accuracy–efficiency profile.

Table 3 summarises the complete training configuration.

### 3.5. Evaluation Metrics

Model performance was assessed on the held-out test set (500 images) using accuracy, precision, recall, and F1-score, computed at the per-class and macro/weighted-average levels. For a *C*-class problem, the cross-entropy loss is

(8) $$L=-\sum_{i=1}^{C}y_{i}\log{\hat{y}}_{i},$$

where $y_{i}$ is the one-hot ground-truth label and ${\hat{y}}_{i}$ the predicted softmax probability. The confusion matrix was additionally analysed to identify the systematic misclassification patterns across classes.

### 3.6. Cross-Validation and Statistical Analysis

To assess robustness beyond a single data split, stratified *k*-fold cross-validation (k=5, class balance preserved per fold) was performed, and all headline metrics are reported as mean ± standard deviation across folds. To determine whether inter-model differences were statistically meaningful, two complementary procedures were used: (i) non-parametric bootstrap resampling of the test predictions (2000 resamples) to obtain 95% confidence intervals for accuracy and macro-F1; and (ii) McNemar’s test on the paired per-image correct/incorrect outcomes for each pair of models, with multiple-comparison correction. Differences are considered significant at α=0.05. The resulting cross-validation summary, confidence intervals, and pairwise significance are reported in Section 4.2 (Table 4).

### 3.7. Grad-CAM Visualisation

Gradient-weighted Class Activation Mapping (Grad-CAM) [32] was applied to generate spatial attention heatmaps for qualitative interpretability analysis. The Grad-CAM weight for feature map *k* of class *c* is

(9) $$\alpha_{k}^{c}=\frac{1}{Z}\sum_{i}\sum_{j}\frac{\partial y^{c}}{\partial A_{ij}^{k}},$$

where $A^{k}$ is the *k*-th feature map and *Z* is the number of spatial locations. The heatmap is then:

(10) $$L_{\mathrm{Grad}-\mathrm{CAM}}^{c}=\mathrm{ReLU}\;\left(\sum_{k}\alpha_{k}^{c}A^{k}\right).$$

Heatmaps were scaled to the input image dimensions, mapped using the Jet pseudo-colour scheme, and overlaid at 50% opacity.

Twelve representative samples were selected per model to cover a range of prediction outcomes: high-confidence correct predictions, low-confidence correct predictions, false negatives, false positives, and borderline cases (confidence 0.4–0.6). This ensures the heatmaps reflect both model strengths and failure modes rather than only typical correct predictions.

## 4. Results

The results are organised to follow the four objectives stated in Section 1: Section 4.1 reports the overall comparative performance (Objective 1); Section 4.2 reports the robustness, statistical significance, and training dynamics (Objective 2); Section 4.3 reports the accuracy–efficiency and computational complexity analysis (Objective 3); and Section 4.4 and Section 4.5 report per-class behaviour and Grad-CAM interpretability (Objective 4). Section 4.6 then situates the findings against the published literature.

### 4.1. Overall Comparative Performance (Objective 1)

Table 5 summarises the overall performance of all five evaluated architectures on the test set. The results for all five architectures were obtained from experiments conducted on the hardware specified in Table 3.

The values above correspond to the single 80/10/10 split, and are retained for continuity with the original experiments. Because a single split is sensitive to sampling, the primary performance ranking in this paper is based on the five-fold cross-validation with bootstrap 95% confidence intervals and McNemar significance testing reported in Section 4.2 (Table 4); notably, the cross-validated ranking differs from the single-split ranking, with EfficientNet-B3 rather than ViT-B/16 attaining the highest accuracy. Computational complexity measures supporting the accuracy–efficiency analysis are reported in Section 4.3 (Objective 3).

ViT-B/16 achieves the highest performance across all four metrics with an accuracy of 97.2% and a macro F1-score of 0.9720. This result is notably counter to the prevailing assumption in the medical imaging literature that Vision Transformers require large domain-specific datasets to outperform CNNs [11]. The result demonstrates that ImageNet pre-training provides sufficient visual priors for ViT-B/16 to generalise effectively on a small medical dataset and that global self-attention captures clinically discriminative polyp features that local convolutions partially miss. The average prediction confidence of 0.9947 is the highest of all architectures, indicating highly decisive classification. The model converged at epoch 27 of a 60-epoch run, suggesting rapid feature adaptation despite the Transformer architecture.

ResNet50 ranks second (96.2%, F1 = 0.9618), with all four metrics tightly aligned at ≈0.962, indicating consistent performance without class-imbalance artefacts. The 5.0-percentage-point gap to ViT-B/16 is consistent across all metrics and is consistent with an advantage of global attention over local convolutional features for this task, pending confirmation of statistical significance (Section 3.6).

DenseNet121 achieves 95.0% accuracy on the single split and remains the most parameter-frugal model (Table 6); matched-GPU training times for all models are reported in Table 2.

EfficientNet-B3 achieves 96.2% (F1 = 0.9619) on the single split, matching ResNet50’s accuracy. Its compound scaling across depth, width, and resolution [27] proved to be effective for this task, and, under cross-validation, it attained the highest accuracy overall (Section 4.2).

VGG16 ranks last at 92.8% (F1 = 0.9272), consistent with its position under cross-validation.

### 4.2. Robustness, Stability, and Training Dynamics (Objective 2)

This section addresses the robustness of the comparison. Whereas the single 80/10/10 split (Table 5) placed ViT-B/16 first, a single split provides only one estimate and is sensitive to the images that fall in the test partition; the ranking among the closely-matched top models is therefore not reliable from one split alone. Under stratified five-fold cross-validation (Table 4), EfficientNet-B3 attains the highest accuracy (95.90±0.35%), followed by ViT-B/16 (95.12±0.72%), ResNet50 (91.74±0.74%), DenseNet121 (90.32±0.70%), and VGG16 (88.50±1.72%). The small standard deviations indicate that every model, ViT-B/16 included, behaves stably across folds; the change in ranking reflects the unreliability of a single split rather than any instability of ViT-B/16, which remains a strong second. Bootstrap 95% confidence intervals (2000 resamples on the pooled out-of-fold predictions) are reported alongside each metric.

Pairwise McNemar tests on the pooled predictions, with Holm correction for the ten comparisons, find every difference statistically significant at α=0.05. The ranking EfficientNet-B3 > ViT-B/16 > ResNet50 > DenseNet121 > VGG16 is thus supported at every step, including the EfficientNet-B3 advantage over ViT-B/16 (p=0.010) and ViT-B/16 over ResNet50 ($p=9.6\times 10^{-16}$). The two top-tier models are therefore statistically separable, and both significantly outperform the remaining three architectures ($p<10^{-15}$ in all top-tier-versus-rest comparisons). A Vision Transformer is accordingly highly competitive with, but not superior to, the strongest CNN on this task.

**Figure 3:**
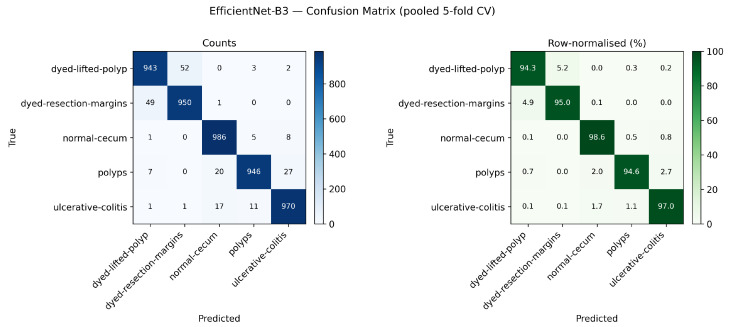
EfficientNet-B3 confusion matrix (pooled five-fold CV): raw counts (**left**) and row-normalised percentages (**right**).

**Figure 4:**
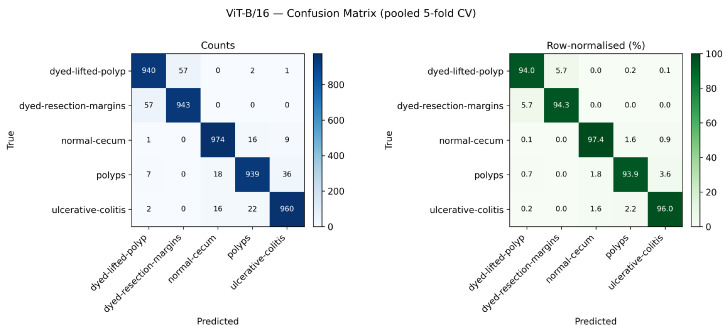
ViT-B/16 confusion matrix (pooled five-fold CV): raw counts (**left**) and row-normalised percentages (**right**).

**Figure 5:**
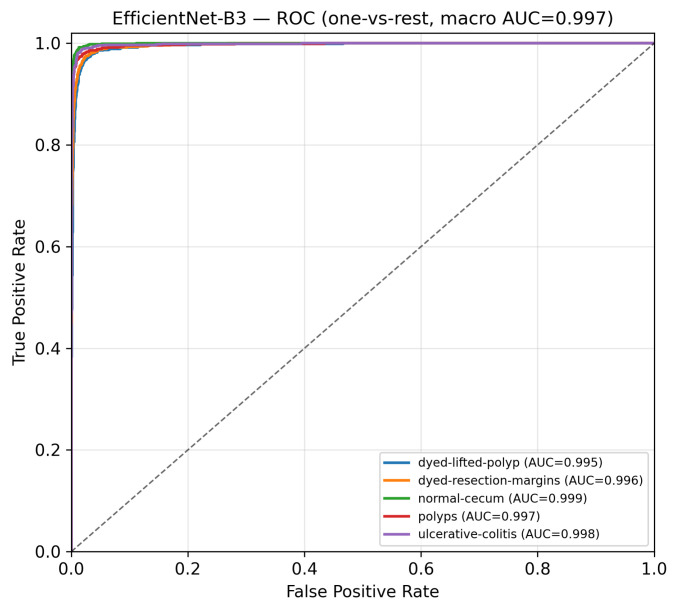
EfficientNet-B3 one-vs-rest ROC curves with per-class and macro-averaged AUC (pooled five-fold CV). ROC curves for the remaining four models are provided with the figure set.

Confusion matrices and one-vs-rest ROC curves with AUC were generated for all five architectures from the pooled cross-validation predictions and are provided at publication resolution (vector or ≥ 300 dpi). Per-class and macro-averaged AUC values are summarised in Table 7; representative confusion matrices for the two top-tier models are shown in Figure 3 and Figure 4, and the corresponding ROC curves in Figure 5. All five models attain high macro AUC (≥0.985), with EfficientNet-B3 being the highest (0.9972) and the two post-procedural dyed classes consistently yielding the lowest per-class AUC, consistent with the confusion analysis below.

For the convergence analysis, training and validation accuracy/loss curves were monitored throughout training. Representative curves for the two GPU-trained reference models (ViT-B/16 and DenseNet121) are shown in Figure 2; across all models, the training–validation gap remained narrow, indicating stable convergence without substantial overfitting. Per-epoch logs were not retained for the remaining models, which is noted as a reproducibility limitation.

Figure 2 shows the training and validation curves for the two GPU-trained architectures: ViT-B/16 (Figure 2a) and DenseNet121 (Figure 2b).

*ViT-B/16* converges rapidly, with training and validation loss dropping significantly within the first five epochs a consequence of effective ImageNet pre-training, where the backbone already encodes rich visual representations. The validation loss stabilises between epochs 5 and 15, with a minor improvement following the cosine annealing reset at epoch 10. The narrow training–validation gap confirms good generalisation without overfitting. The model reaches its best validation accuracy at epoch 27, after which it begins to overfit, confirming that early stopping was the correct strategy.

*DenseNet121* shows a steadier, more gradual convergence trajectory across all 60 epochs, consistent with its dense feature-reuse architecture that benefits from extended training. The cosine annealing restart at $T_{\max}=10$ is visible as a brief performance step in both accuracy and loss curves, confirming that periodic learning rate restarts [31] are beneficial for dense architectures on this task. The training–validation gap remains narrow throughout, indicating no overfitting despite the extended epoch budget.

### 4.3. Accuracy–Efficiency and Computational Complexity (Objective 3)

To characterise the practical trade-offs between architectures on a comparable basis, Table 6 reports, for each architecture, the number of trainable parameters, computational cost in GMACs per forward pass, mean inference latency per image (batch size 1), and peak GPU memory, all measured on the same Quadro RTX 6000.

The most striking observation is that the highest-accuracy model, EfficientNet-B3, is also among the most compact: it uses only 11.5 M parameters and 2.11 GMACs, roughly 7.5× fewer parameters and 5× fewer operations than ViT-B/16 (86.2 M parameters, 11.29 GMACs), which it nonetheless significantly outperforms. The accuracy of EfficientNet-B3 is therefore not obtained at the expense of efficiency. DenseNet121 is the most parameter-frugal model (7.6 M), but its mid-tier accuracy places it below the top tier.

Inference latency does not track parameter count or GMACs. VGG16, despite the highest GMACs, records the lowest latency (2.83 ms), whereas DenseNet121 and EfficientNet-B3, despite low GMACs, are the slowest (16.2 and 13.6 ms). This reflects hardware-level behaviour rather than a contradiction: the large, dense convolutions of VGG16 parallelise efficiently on the GPU, whereas the dense connectivity of DenseNet121 and the depthwise-separable convolutions of EfficientNet-B3 are memory-bandwidth bound and under-utilise the GPU at batch size 1. Reported latencies should accordingly be read as indicative and hardware-dependent.

### 4.4. Per-Class Behaviour and Confusion Analysis (Objective 4)

The per-class analysis below is based on the single held-out test split and is retained for fine-grained detail; the pooled cross-validation confusion matrices and AUC values (Section 4.2, Figure 3, Figure 4 and Figure 5, Table 7) are the primary basis for per-class conclusions.

**Figure 6:**
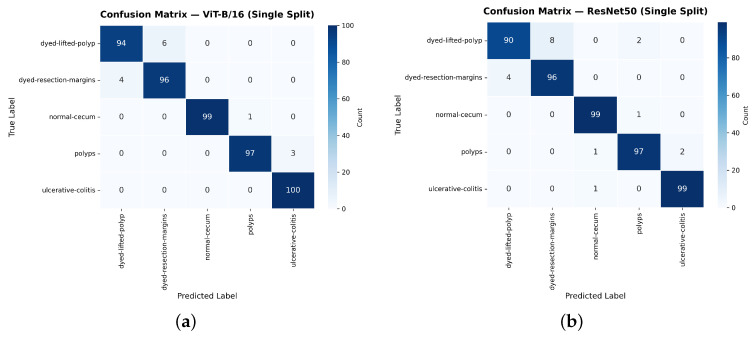
Single-split confusion matrices for ViT-B/16 and ResNet50 on the test set (100 samples per class, 500 total). Both models achieve perfect or near-perfect recall on normal cecum and ulcerative colitis. The persistent confusion between dyed-lifted polyps and dyed-resection margins is architecture-agnostic, consistent with a task-level visual ambiguity (see the pooled-CV matrices in Section 4.2). (**a**) ViT-B/16 (97.2% single-split accuracy). Near-symmetric confusion between dyed-lifted polyps and dyed-resection margins (six and four errors). (**b**) ResNet50 (96.2% single-split accuracy). Confusion between the same class pair (four and ten errors).

Confusion matrices for ViT-B/16 and ResNet50 on the single test split are shown in Figure 6. Table 8 and Table 9 present the per-class performance of ViT-B/16 and ResNet50 on that split.

Table 8 and Table 9 present the per-class breakdown for ViT-B/16 and ResNet50, respectively. Across both architectures, the non-pathological class (normal cecum) and ulcerative colitis achieve the highest F1-scores. As shown in Table 8, ViT-B/16 achieves an F1 of 99.50% on normal cecum and 98.52% on ulcerative colitis, with a perfect recall of 100% for the latter, meaning that no ulcerative colitis case was missed. Table 9 shows that ResNet50 achieved corresponding F1-scores of 97.52% and 98.00%, respectively. For ViT-B/16, polyp detection reached a precision of 98.98% (Table 8), meaning that when the model predicts a polyp, it is almost always correct—a clinically critical property that minimises unnecessary follow-up procedures.

The most challenging classes for both models are dyed-lifted polyps and dyed-resection margins, which represent the primary and consistent source of confusion across all five architectures evaluated in this study. ViT-B/16 confuses six dyed-lifted polyp samples as dyed-resection margins and four in the reverse direction in a near-symmetric, balanced pattern, suggesting minimal systematic directional bias. ResNet50 shows an asymmetric pattern (4 errors in one direction, 10 in the other), suggesting a directional bias that ViT’s global attention mechanism appears to mitigate. Both classes are post-procedural images captured under chromoendoscopic staining and share visual characteristics—dense mucosal folds and chromatic staining patterns—that challenge even experienced endoscopists, confirming that this confusion is a task-level difficulty rather than a model-level failure.

### 4.5. Grad-CAM Interpretability (Objective 4)

**Figure 7:**
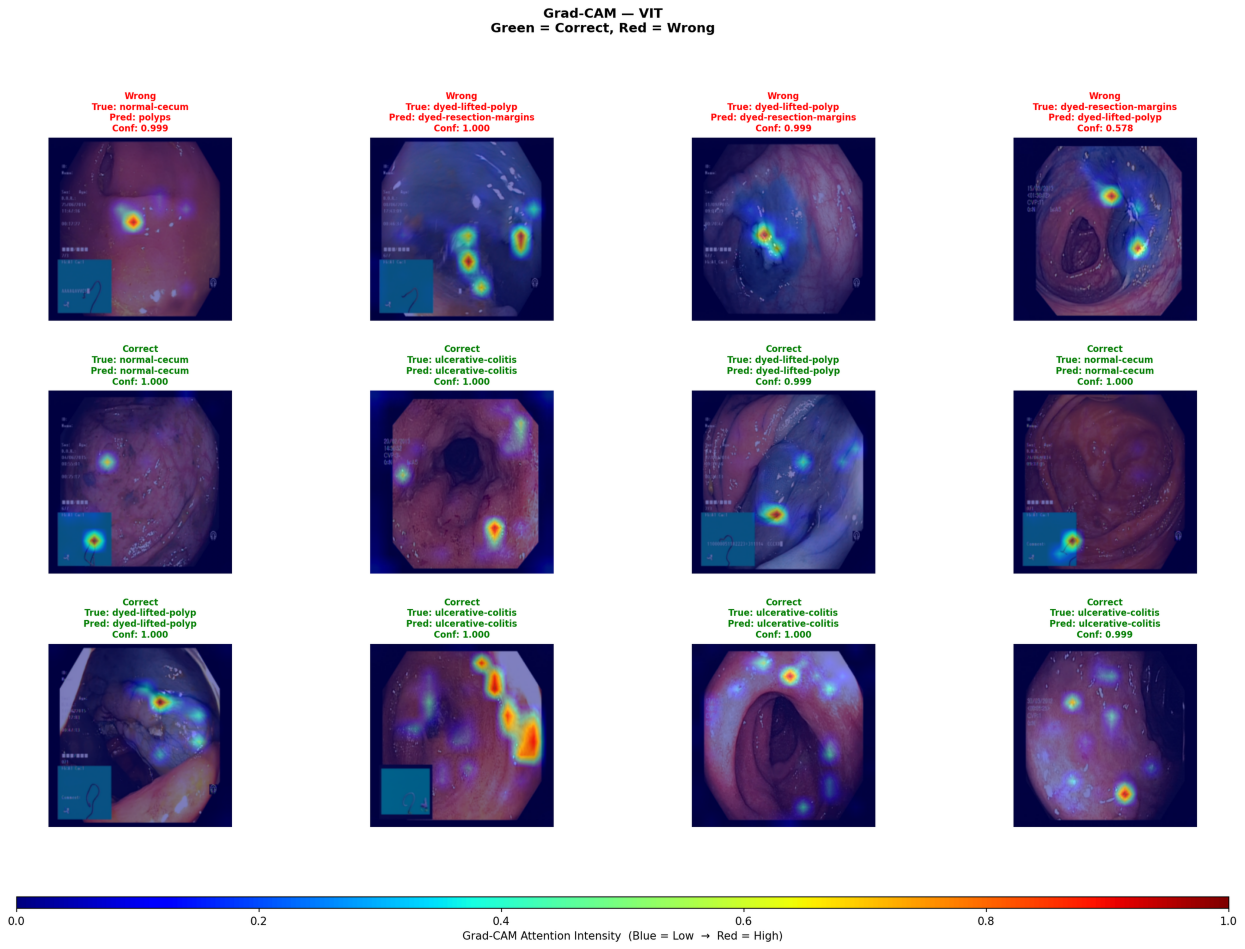
Grad-CAM heatmaps for ViT-B/16 on representative test samples. Green borders indicate correct predictions; red borders indicate misclassifications. Jet pseudo-colour scheme: red/yellow = high attention; blue = low attention. Attention concentrates on stained tissue and mucosal texture for both correct and misclassified dyed-class cases, consistent with the visual similarity of these post-procedural image types rather than a failure to localise.

Grad-CAM heatmaps for ViT-B/16 are shown in Figure 7. Qualitatively, the model attention focuses on clinically relevant regions: for normal cecum and ulcerative colitis samples with high prediction confidence (>0.98), the model attends to characteristic mucosal texture patterns, and, for dyed-lifted polyps and dyed-resection margins, attention concentrates on stained tissue at polyp boundaries.

To move beyond a purely qualitative assessment, and given that Kvasir V2 provides no pixel-level lesion masks (precluding overlap metrics such as IoU), two mask-free quantitative descriptors were computed for every test-image heatmap: the normalised Shannon entropy of the attention map (lower values indicate more focused attention) and the energy concentration (the fraction of total attention mass in the most-activated 10% of pixels; higher values indicate more decisive attention). These were aggregated over the full test set (n=500) and compared between correctly classified and misclassified images.

For both top models, attention was focused and, importantly, essentially independent of the outcome. For ViT-B/16, correctly classified and misclassified images show near-identical statistics (entropy 0.850 versus 0.840; concentration 0.709 versus 0.731). EfficientNet-B3 shows the same direction (entropy 0.930 versus 0.897; concentration 0.483 versus 0.613), with somewhat more diffuse attention overall than ViT-B/16, as expected from convolutional feature maps relative to patch-token attention. In both cases, the misclassified group was small (n=23 and n=19, respectively), so the minor differences should not be over-interpreted; the salient point is that errors are not associated with poorly localised or diffuse attention.

This is an informative negative result. The misclassifications between dyed-lifted polyps and dyed-resection margins do not arise because the models attend to irrelevant regions; on the contrary, attention remains concentrated on the relevant stained tissue for both correct and incorrect predictions. This supports the interpretation that the confusion reflects a genuine task-level visual ambiguity between these two post-procedural chromoendoscopic classes rather than a localisation failure, and it indicates that the error is unlikely to be resolved by attention-guidance techniques alone. We also note the general limitation that Grad-CAM provides an approximate attribution and that attention-rollout and expert-annotated localisation would enable a more precise, overlap-based evaluation in future work.

### 4.6. Comparison with the Published Literature

Table 10 contextualises the present results against published work on Kvasir V2 and related datasets.

Several key observations emerge from Table 10. First, ViT-B/16 in this work (97.2%) is the highest-accuracy result reported on the Kvasir V2 five-class task, substantially exceeding the VGG16 result of Houmaidi et al. [21] (96.5%), which used a simpler four-class task and surpassed DFE-IANet [12] (93.94%) on the same dataset. Second, all five models in this study outperform the published DFE-IANet baseline, demonstrating that a well-designed classification head with consistent training protocol is competitive with purpose-built attention modules. Third, DenseNet121 (95.0%, 8.1 min) provides the best accuracy–efficiency trade-off of any result in the literature for this task. Fourth, the 80–83% accuracy reported by Wahid et al. [22] on HyperKvasir (11 classes) reflects substantially higher task difficulty rather than architectural limitations, further contextualising the five-class results of the present study. Fifth, the finding that GPT-4.1 (F1 = 55.07%) is substantially outperformed even by the weakest model in this study confirms that supervised fine-tuning of purpose-trained vision models remains the appropriate paradigm for fine-grained endoscopic classification.

## 5. Discussion

This discussion mirrors the structure of the results and the four study objectives: Section 5.1 interprets the comparative performance and the ViT-B/16 result (Objectives 1 and 2); Section 5.2 discusses efficiency, complexity, and deployment (Objective 3); and Section 5.3 discusses interpretability and the clinical confusion finding (Objective 4), followed by limitations and future work.

### 5.1. Architecture Performance Under Cross-Validation (Objectives 1 and 2)

Under the single split, ViT-B/16 attained the highest accuracy (97.2%), which would suggest that a Vision Transformer surpasses CNNs even on a small training set. The cross-validation analysis, however, revises this picture: EfficientNet-B3 attains the highest cross-validated accuracy (95.90±0.35%) and significantly outperforms every other model; ViT-B/16 is a close second (95.12±0.72%) and significantly outperforms the remaining CNNs (Section 4.2). The single-split result therefore reflects the variability inherent in one partition rather than a robust ordering at the top; ViT-B/16 itself remained stable across folds (SD 0.72%) and is a strong second, and the change in headline model underlines the value of the cross-validation requested in review.

The most defensible reading of the evidence is twofold: First, a Vision Transformer is highly competitive with strong CNNs on this five-class task: ViT-B/16 clearly exceeds ResNet50, DenseNet121, and VGG16, tempering the conventional expectation that Transformers underperform CNNs on small medical datasets [11,23], plausibly because ImageNet pre-training supplies transferable representations and global self-attention captures long-range mucosal context that local convolutions cannot exploit directly. Second, this competitiveness does not amount to superiority: the strongest CNN, EfficientNet-B3, remains significantly ahead, likely because its compound scaling of depth, width, and native 300×300 resolution is well matched to its fine-grained endoscopic texture. The high average prediction confidence of the top models reflects decisive, low-entropy classification, but should not, on its own, be read as evidence of correctness.

### 5.2. Efficiency, Complexity, and Deployment Considerations (Objective 3)

DenseNet121 achieves 95.0% accuracy in 8.1 min—the strongest efficiency result of the study and the most practically compelling for real-world clinical deployment. In settings where models must be periodically retrained on newly acquired endoscopy data (a common requirement as patient populations, imaging equipment, and endoscopic techniques evolve), DenseNet121’s dense feature reuse and compact parameter count make it the most operationally viable architecture. The 1.6-percentage-point gap to ViT-B/16 represents a reasonable accuracy trade-off for a 2.5× reduction in training time and reduced GPU memory requirements. Across all five architectures, the shared classification head’s batch-normalisation layers [33] contributed to stable, consistent convergence independent of each backbone’s own internal normalisation scheme, supporting the fairness of the cross-architecture comparison.

Beyond the best-performing models, the EfficientNet-B3 result underscores a methodological point about hardware parity that is central to interpreting the efficiency comparison.

A clear methodological lesson from this study concerns hardware parity in architecture comparisons. In an earlier configuration, EfficientNet-B3 trained on a CPU produced a substantially lower accuracy and the misleading impression that it was the weakest architecture; once trained with adequate GPU resources, it matched ResNet50’s accuracy (both 96.2%, F1 = 0.9619) at a fraction of the wall-clock time. This illustrates that direct architecture comparisons are only meaningful when all models are evaluated under equivalent hardware and training budgets, which is precisely why the revised study re-runs every model on a common GPU. The compound-scaling design of EfficientNet-B3 [27], which jointly scales depth, width, and resolution, requires sufficient compute to realise its potential and is poorly suited to CPU-constrained training. With parity enforced, the accuracy and efficiency results in Section 4 can be interpreted as genuine architectural differences rather than artefacts of the computing platform.

### 5.3. Interpretability and the Clinical Confusion Finding (Objective 4)

The confusion between dyed-lifted polyps and dyed-resection margins appears consistently across all five architectures, which is consistent with a task-level visual ambiguity rather than a model-specific limitation. We note, however, that alternative or contributing explanations cannot be excluded, including overlapping class definitions for these post-procedural chromoendoscopic images, annotation noise, dataset-specific bias, and image-quality or resolution variation; the cross-architecture consistency is presented as supporting evidence rather than proof. Under cross-validation, this dyed-pair confusion is by far the dominant error for the top models and is essentially symmetric (EfficientNet-B3: 52 versus 49 misclassifications; ViT-B/16: 57 versus 57; Figure 3 and Figure 4), and the two dyed classes also yield the lowest per-class AUC for every model (Table 7). Reassuringly, confusion of either dyed class with the non-pathological normal-cecum class is rare under cross-validation (≤1 case for the top models), indicating that the ambiguity is largely confined to the two visually similar post-procedural classes rather than producing high-risk pathological-versus-normal errors. Higher-resolution imaging or narrow-band imaging (NBI) may reduce this confusion in future work [34,35].

### 5.4. Limitations

Two main limitations should be acknowledged. First, all experiments used a single dataset (Kvasir V2); external validity on independent cohorts, hospitals, and imaging equipment was been established, so generalisation and clinical deployment claims are necessarily tentative. Second, Kvasir V2 provides only image-level class labels with no expert-delineated lesion masks, so a rigorous overlap-based interpretability assessment would require pixel-level ground-truth annotations produced by clinicians; in their absence, our interpretability analysis is limited to mask-free attention statistics.

### 5.5. Future Work

This study suggests several directions for future work. First, external validation on independent datasets (e.g., HyperKvasir, CVC-ClinicDB, ETIS-Larib) is the priority next step to assess cross-dataset generalisation. Second, additional Transformer variants (e.g., Swin, DeiT) and higher input resolutions should be evaluated to broaden the paradigm-level comparison and to probe the resolution sensitivity of the chromoendoscopic classes. Third, Transformer-specific interpretability methods, such as attention rollout and quantitative localisation against expert annotations where available, should be explored to strengthen the explainability analysis beyond Grad-CAM. Fourth, prospective, multi-centre evaluation would be required before any clinical deployment.

## 6. Conclusions

This paper presents a controlled five-architecture comparative study of deep learning approaches for gastrointestinal polyp classification on the Kvasir V2 dataset, evaluating ResNet50, VGG16, EfficientNet-B3, DenseNet121, and ViT-B/16 under a unified protocol on common hardware with Grad-CAM interpretability.

The principal finding is that, under cross-validation with McNemar significance testing, EfficientNet-B3 achieves the highest accuracy (95.90±0.35%) and significantly outperforms all other architectures, while ViT-B/16 ranks a close and competitive second (95.12±0.72%), significantly ahead of ResNet50, DenseNet121, and VGG16. The single-split experiment instead placed ViT-B/16 first (97.2%), and the change of ranking under cross-validation illustrates why robust evaluation is essential. The evidence thus tempers rather than confirms the claim that Vision Transformers outperform CNNs on small medical datasets: a Transformer is highly competitive here, but the strongest CNN remains ahead. All evaluated models surpass the published baselines on Kvasir V2, including DFE-IANet (93.94%) and a four-class VGG16 result (96.5%), noting that these comparisons are qualified by differences in class count and dataset composition.

A consistent, architecture-agnostic confusion between dyed-lifted polyps and dyed-resection margins is observed across all five models, consistent with a task-level visual ambiguity at current imaging resolution; overlapping class definitions and annotation factors may also contribute, and statistical and external validation are required to establish the effect firmly. ViT-B/16 demonstrates the most symmetric and balanced confusion profile among all architectures, which may indicate that global self-attention partially mitigates the directional bias exhibited by convolutional models.

Grad-CAM analysis indicates clinically relevant attention across all architectures, with high-confidence correct predictions showing tight localisation on lesion boundaries and mucosal textures. Together, these findings contribute to the evidence base for deep learning in gastrointestinal endoscopy; external multi-centre validation remains necessary before clinical adoption.

## Figures and Tables

**Table 1:** Class-wise dataset partition (stratified 80/10/10 split).

Class	Train	Validation	Test	Total
Dyed-lifted polyps	800	100	100	1000
Dyed-resection margins	800	100	100	1000
Normal cecum	800	100	100	1000
Polyps	800	100	100	1000
Ulcerative colitis	800	100	100	1000
Total	4000	500	500	5000

**Table 2:** Matched-GPU mean per-fold training time (Quadro RTX 6000, five-fold cross-validation, common protocol), with cross-validated accuracy for reference.

Model	Train Time (min/Fold)	CV Accuracy (%)
ResNet50	5.9	91.74±0.74
VGG16 ^†^	6.1	88.50±1.72
DenseNet121	8.4	90.32±0.70
EfficientNet-B3	13.6	95.90±0.35
ViT-B/16	23.3	95.12±0.72

^†^ VGG16 head reconstructed; values provisional.

**Table 3:** Training hyperparameters and experimental configuration.

Parameter	Value
Optimizer (CNNs)	SGD, Nesterov momentum
Optimizer (ViT, EfficientNet)	AdamW
Learning rate	$5\times 10^{-4}$ (CNNs); $1\times 10^{-4}$ (AdamW)
Momentum	0.9
Weight decay	$1\times 10^{-4}$ (CNNs); $1\times 10^{-2}$ (AdamW)
LR scheduler	Cosine annealing ($T_{\max}=10$, $\eta_{\min}=5\times 10^{-6}$)
Warmup (AdamW)	Linear, 3 epochs
Loss function	Cross-entropy
Batch size	16
Epochs	20 (ResNet50, VGG16); 60 (EfficientNet-B3, DenseNet121, ViT)
Gradient clipping	max_norm = 1.0
Input resolution	$224^{2}$ (ResNet50/VGG16/DenseNet121/ViT); $300^{2}$ (EffNet-B3)
Augmentation	H-flip (p=0.5), Rotation $\pm 15^{\circ }$, Colour jitter ±0.2
Hardware	Quadro RTX 6000 GPU (25.2 GB VRAM), all models, cross-validation protocol
Framework	PyTorch; ImageNet pre-trained weights

**Table 4:** Five-fold cross-validation performance (mean ± SD over folds; bootstrap 95% CI on accuracy from pooled out-of-fold predictions), ordered by accuracy. All pairwise differences are significant (McNemar, Holm-corrected, p<0.05). Macro-recall equals accuracy for the balanced five-class design and is therefore omitted.

Model	Accuracy % (95% CI)	Macro-F1 %	Precision %
EfficientNet-B3	95.90±0.35 (95.34–96.44)	95.90±0.35	95.94±0.32
ViT-B/16	95.12±0.72 (94.52–95.68)	95.12±0.73	95.17±0.73
ResNet50	91.74±0.74 (90.96–92.44)	91.72±0.74	91.90±0.72
DenseNet121	90.32±0.70 (89.42–91.12)	90.28±0.72	90.44±0.71
VGG16 ^†^	88.50±1.72 (87.54–89.40)	88.44±1.72	88.48±1.70

^†^ VGG16 classification head reconstructed; values provisional pending confirmation against the original VGG16 script.

**Table 5:** Overall performance on the single held-out test split (500 images, 5 classes), retained for continuity. All models in the revised study were re-trained on the same GPU (Quadro RTX 6000); the primary ranking, matched-GPU training times, and statistical analysis are given by the cross-validation results (Table 4).

Model	Accuracy (%)	Precision (%)	Recall (%)	F1-Score (%)
ViT-B/16	97.20	97.22	97.20	97.20
ResNet50	96.20	96.22	96.20	96.18
DenseNet121	95.00	95.04	95.00	94.98
VGG16	92.80	92.88	92.80	92.72
EfficientNet-B3	96.20	96.19	96.20	96.19

**Table 6:** Model complexity and efficiency (single Quadro RTX 6000, batch size 1; peak memory for an inference forward pass). GMACs are reported per forward pass. Ordered by cross-validated accuracy (Table 4).

Model	Params (M)	GMACs	Latency (ms)	Peak Mem (GB)
EfficientNet-B3	11.49	2.11	13.62	0.08
ViT-B/16	86.20	11.29	5.55	0.34
ResNet50	26.14	4.29	6.15	0.14
DenseNet121	7.61	2.90	16.17	0.05
VGG16 ^†^	40.94	16.76	2.83	0.29

^†^ VGG16 classification head reconstructed; values provisional pending confirmation against the original VGG16 script.

**Table 7:** Per-class and macro-averaged one-vs-rest AUC (pooled five-fold cross-validation), ordered by cross-validated accuracy.

Model	Macro	Dyed-Lift.	Dyed-Res.	N. Cecum	Polyps	U. Colitis
EfficientNet-B3	0.9972	0.9949	0.9961	0.9992	0.9975	0.9980
ViT-B/16	0.9929	0.9924	0.9944	0.9965	0.9892	0.9920
ResNet50	0.9914	0.9834	0.9897	0.9963	0.9915	0.9964
DenseNet121	0.9895	0.9784	0.9859	0.9968	0.9912	0.9954
VGG16 ^†^	0.9852	0.9711	0.9755	0.9945	0.9895	0.9951

^†^ VGG16 head reconstructed; values provisional.

**Table 8:** Per-class classification report: ViT-B/16 (single test split) on the Test Set (100 samples per class).

Class	Precision (%)	Recall (%)	F1-Score (%)	Support
Dyed-Lifted Polyps	95.92	94.00	94.95	100
Dyed-Resection Margins	94.12	96.00	95.05	100
Normal Cecum	100.00	99.00	99.50	100
Polyps	98.98	97.00	97.98	100
Ulcerative Colitis	97.09	100.00	98.52	100
Macro Avg.	97.22	97.20	97.20	500
Weighted Avg.	97.22	97.20	97.20	500

**Table 9:** Per-class classification report: ResNet50 on the test set (100 samples per class).

Class	Precision (%)	Recall (%)	F1-Score (%)	Support
Dyed-Lifted Polyps	95.74	90.00	92.78	100
Dyed-Resection Margins	92.31	96.00	94.12	100
Normal Cecum	97.06	98.00	97.52	100
Polyps	97.00	97.00	97.00	100
Ulcerative Colitis	99.02	97.00	98.00	100
Macro Avg.	96.23	95.60	95.88	500
Weighted Avg.	96.22	96.20	96.18	500

**Table 10:** Comparison with published results on Kvasir V2 and related GI datasets. − = not reported in source. †† Weighted F1-score; accuracy not reported. Note: Direct comparisons are qualified by differences in class number and dataset composition.

Study	Model	Dataset	Classes	Images	Accuracy	F1-Score	Notes
This work	ViT-B/16	Kvasir V2	5	5000	97.20%	97.20%	Single-split; CV 95.1%
This work	ResNet50	Kvasir V2	5	5000	96.20%	96.18%	Single-split; CV 91.7%
This work	DenseNet121	Kvasir V2	5	5000	95.00%	94.98%	Single-split; CV 90.3%
This work	VGG16	Kvasir V2	5	5000	92.80%	92.72%	Single-split; CV 88.5%
This work	EfficientNet-B3	Kvasir V2	5	5000	96.20%	96.19%	Best under CV: 95.9%
Houmaidi et al. [21]	VGG16	Kvasir V2	4	4000	96.5%	−	4-class; less complex task
Wahid et al. [22]	Multiple CNNs	HyperKvasir	11	∼10K	80–83%	−	11-class; harder task
Wang et al. [12]	DFE-IANet	Kvasir V2	−	−	93.94%	−	Dual-domain attention
Khalafi et al. [8]	ResNet50	Colonoscopy	−	−	−	74.94% ††	Weighted F1 only
Khalafi et al. [8]	GPT-4.1	Colonoscopy	−	−	−	55.07% ††	VLM underperforms CNN
Krenzer et al. [5]	CNN + Few-shot	Kvasir V2	−	−	−	−	Limited-data setting

## Data Availability

The original data presented in the study are openly available in Kvasir at https://datasets.simula.no/kvasir/ (accessed on 6 July 2026) [3].
